# Interfacial Enzymes Enable Gram-Positive Microbes to Eat Fatty Acids

**DOI:** 10.3390/membranes13040423

**Published:** 2023-04-10

**Authors:** Christopher D. Radka

**Affiliations:** Department of Infectious Diseases, St. Jude Children’s Research Hospital, Memphis, TN 38105, USA; christopher.radka@stjude.org; Tel.: +1-901-595-5616

**Keywords:** interfacial enzymes, fatty acid metabolism, peripheral membrane proteins, membrane binding, mechanism

## Abstract

Exogenous fatty acid (eFA) activation and utilization play key roles in bacterial physiology and confer growth advantages by bypassing the need to make fatty acids for lipid synthesis. In Gram-positive bacteria, eFA activation and utilization is generally carried out by the fatty acid kinase (FakAB) two-component system that converts eFA to acyl phosphate, and the acyl-ACP:phosphate transacylase (PlsX) that catalyzes the reversible conversion of acyl phosphate to acyl–acyl carrier protein. Acyl–acyl carrier protein is a soluble format of the fatty acid that is compatible with cellular metabolic enzymes and can feed multiple processes including the fatty acid biosynthesis pathway. The combination of FakAB and PlsX enables the bacteria to channel eFA nutrients. These key enzymes are peripheral membrane interfacial proteins that associate with the membrane through amphipathic helices and hydrophobic loops. In this review, we discuss the biochemical and biophysical advances that have established the structural features that drive FakB or PlsX association with the membrane, and how these protein–lipid interactions contribute to enzyme catalysis.

## 1. Introduction

### 1.1. Protein–Membrane Association

The bacterial cytoplasmic membrane envelopes the cell and separates the cytosol from the extracellular environment. The cytoplasmic membrane is loaded with proteins that functionalize the membrane for a variety of processes including energy generation, import and export, and signaling. Bacterial membranes can be made up of as much as 50% proteins, thus demonstrating their importance to the membrane composition [1].

Integral membrane proteins are completely embedded in the hydrophobic core of the membrane and span the lipid bilayer. The membrane-spanning segments of these proteins are rich in hydrophobic amino acids whose side chains interact with the hydrophobic core and transmembrane structures and generally consist of one or multiple α-helices or a rolled-up β-sheet (i.e., β-barrel). Hydrophilic segments on either side of the bilayer enable integral membrane proteins to recognize and respond to soluble intracellular or extracellular factors. Monotopic proteins are a subset of integral membrane proteins that are permanently fixed to one leaflet of the membrane through amphipathic α-helices or hydrophobic loops.

Lipid-anchored proteins contain covalently attached lipids or fatty acids. The hydrophobic segment of the lipid or fatty acid is embedded in one of the membrane leaflets and anchors the water-soluble protein to the bilayer, whereas the polypeptide chain typically plays a negligible role in entering the bilayer. Both Gram-positive and Gram-negative bacteria have lipoproteins with covalently attached fatty acids on their amino termini [2,3]. Bacterial lipoproteins are potent agonists that stimulate the immune system, but when the fatty acids are hydrolyzed from the lipoprotein, the immunostimulatory property of the deacylated protein is inactivated [4].

### 1.2. Interfacial Enzymes

Peripheral membrane proteins indirectly associate with the membrane through noncovalent interactions with integral or lipid-anchored proteins, or directly through electrostatic or ionic interactions with the bilayer lipid head groups. Two of the most common structural elements that drive peripheral membrane protein association with the membrane are amphipathic helices and hydrophobic loops [5,6,7,8]. Interfacial enzymes whose substrates are lipids must bind to the membrane surface to carry out their function. Interfacial enzymes are soluble proteins that gather on a membrane surface by interfacial adsorption. This process can have the effect of concentrating and stabilizing protein on the membrane, and hyper-activating the enzyme to enhance its specific activity (also referred to as interfacial activation). Interfacial adsorption is crucial for interfacial enzymes because their hydrophobic substrates are water-insoluble and partition into the membrane. Therefore, the enzyme must interact with the membrane to entice the substrate into its active site. Membrane binding induces a conformational change that makes a productive enzyme-substrate complex and therefore enables extraction of the substrate from the membrane. Lipases are a well-studied example of water-soluble enzymes that reversibly bind the membrane and catalyze a hydrolysis reaction at the lipid–water interface [9].

Since they are soluble, interfacial enzymes are readily used to catalyze unnatural reactions to modify hydrophobic substrates and analogs that are delivered by artificial bilayers (i.e., liposomes and vesicles), detergents, or carrier proteins (i.e., albumin) as surrogates for natural membranes. However, natural membranes are complex, having diverse lipid compositions, and are integral for membrane-associated proteins that may contribute to the overall interfacial enzyme–membrane binding event. It is unclear if detergents or protein carrier membrane surrogates elicit the full physiological change in interfacial enzymes that natural membranes do. Thus, caution should be used when inferring natural membrane binding properties from studies using membrane surrogates, particularly those with detergent or carrier proteins. Artificial bilayers are an optimal reagent for biophysical studies of the mode of membrane binding (i.e., insertion depth, angular orientation, electrostatic interaction).

### 1.3. Membrane Binding Experimental Methods

A common biophysical technique to measure lipid affinity for interfacial enzymes is surface plasmon resonance (SPR) [10]. Lipids can be immobilized to an SPR L1 carboxydextran-coated chip surface, and the enzyme of interest can be injected in increasing concentrations to measure binding affinity. Interfacial enzymes adsorb to the lipid and then desorb under denaturing conditions (i.e., sodium hydroxide wash) to reuse the immobilized lipids and repeat the binding process at another enzyme concentration or buffer composition. Lipids are removed from the chip using detergents to regenerate the L1 chip with a new lipid composition and take advantage of the reversibility of the immobilization technique.

Other biophysical tools used to study lipid binding include nuclear magnetic resonance (NMR) and electron paramagnetic resonance (EPR). EPR focuses on the interaction of an external magnetic field with an unpaired electron spin in a molecule, while NMR focuses on the interaction of an external magnetic field with isotopic nuclei of the individual atom [11]. Site-directed spin labeling of amino acid side chains in EPR experiments enables the determination of properties or protein–membrane interactions, such as the topology of the protein with respect to the membrane, and information about local secondary structure in the membrane and degree of membrane insertion [11,12]. In NMR, isotropic chemical shifts and spin exchange signals are converted to torsion angles and interatomic distances, and dipolar couplings and anisotropic chemical shifts are converted to bond orientation restraints to provide dynamic atomic information about the location of amino acids [13]. NMR signals are sensitive to local environments and can be used to study the structure–activity correlations of proteins in detergent micelles versus detergent-free lipids [13], the binding events of ligand-induced conformational rearrangements, and the effect of allostery on the equilibrium of conformational exchange [14].

Molecular dynamics (MDs) is a useful computational technique for hypothesis generation through visualization of hypothetical membrane binding. MD simulations enable identification of possible key residues that may be responsible for tight binding and candidates for mutagenesis and validation of the simulations. During MD simulations, a substantial portion of the computational resources are spent on simulating the dynamics of lipid acyl chains, which are not expected to play a major role in interfacial adsorption. To reduce this computational cost and shorten simulation times for higher throughput, the acyl chains can be truncated and the empty space filled with an oil layer of fewer atoms (“accelerated membrane models” or “highly mobile membrane mimetic”) [15]. This method has been validated to be reliable and yields results comparable to simulations with full acyl chains (“full membrane/full lipid”) [16] and will be a useful tool to study the mechanisms of interfacial enzyme–membrane binding.

## 2. Bacterial Fatty Acid Metabolism

Bacterial fatty acid synthesis (FASII) is an essential energy-intensive process that produces the fatty acids required for lipid synthesis and membrane biogenesis [17]. Discrete monofunctional FASII enzymes catalyze the activation of short chain acyl-coenzyme A (acyl-CoA) to acyl–acyl carrier protein (acyl-ACP), which undergoes successive rounds of condensation, reduction, dehydration, and reduction to extend the acyl chain of acyl-ACP by two carbons with each elongation cycle [18]. Fatty acids are insoluble molecules that partition in the membrane compartment of cells and are linked to CoA and ACP for solubility to make them compatible with the soluble FASII enzymes. Nature has evolved a repertoire of antibiotics inhibiting different aspects of FASII, hence validating these enzymes as potential targets for new antibiotic discovery and development [19]. FASII inhibitors also have clinical potential, shown by novel therapeutic candidates advancing through clinical trials, with encouraging antimicrobial efficacy [20,21]. Some bacteria can acquire exogenous fatty acids (eFAs) from their environment and bypass the need to expend energy for de novo synthesis of substrates for membrane biogenesis. eFA can be acquired as free unesterified fatty acids [22] (although this form is generally in low abundance), or extracellular lipases can hydrolyze esterified fatty acids from abundant host lipids and liberate monomeric fatty acids [23]. eFA acquisition has gained attention as a possible FASII inhibition resistance mechanism in the treatment of Gram-positive pathogens, with a major focus on *Staphylococcus aureus* [24,25,26], although heterogeneity in how bacteria eat eFA suggests the viability of FASII inhibition as an antimicrobial strategy may be pathogen-specific [19,27]. *S. aureus* is a leading cause of skin infection and leading cause of death from antibiotic-resistant infection [28], highlighting the need for new therapies in the clinic. Studying the eFA acquisition pathway may uncover new features that can be inhibited and yield novel compounds to enhance the efficacy of FASII inhibitors against many Gram-positive organisms. To this end, this review will discuss advancements made in understanding how Gram-positive bacteria eat eFA.

### 2.1. Bacterial Phospholipid Synthesis

**Figure 1:**
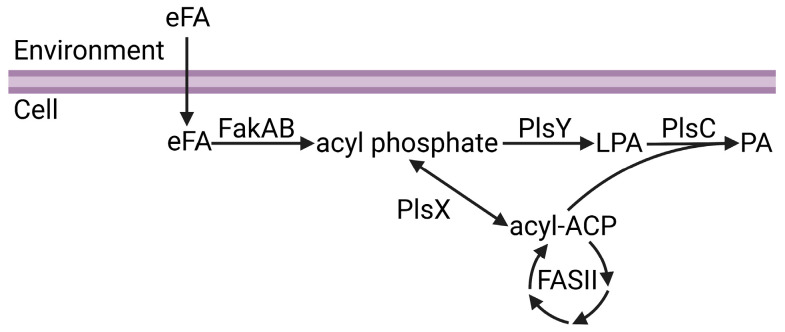
Channeling of exogenous fatty acids (eFA) into phospholipid synthesis. eFA enters the cell and is activated to acyl phosphate by the FakAB two-component system. Acyl phosphate can either be utilized by PlsY to make lysophosphatidic acid (LPA) or converted to acyl-ACP by PlsX. Acyl-ACP can be modified through bacterial fatty acid biosynthesis (FASII) or utilized with LPA by PlsC to make phosphatidic acid (PA). Figure created using BioRender.com.

**Figure 2:**
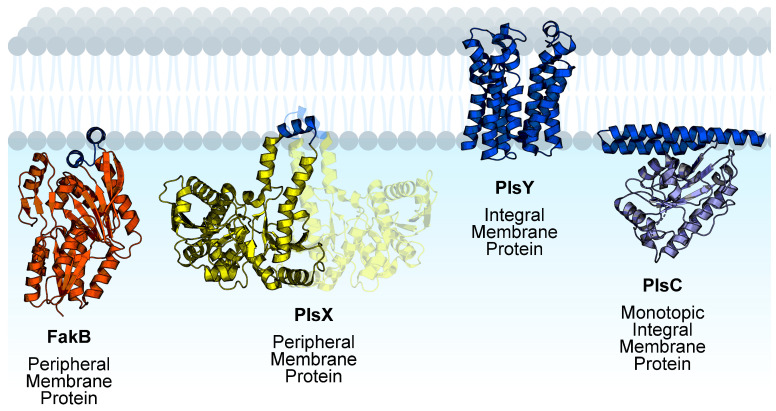
Membrane proteins channel exogenous fatty acids to phospholipid synthesis in Gram-positive bacteria. FakB, PlsY, and PlsC are monomeric proteins. PlsX is dimeric and the opposite protomer is shown with transparency. Membrane-binding domains are shown in blue and soluble domains are shown in orange (FakB), yellow (PlsX), or purple (PlsC). The structures shown are *Staphylococcus aureus* FakB1 (PDB ID:6MH9) [16], *Bacillus subtilis* PlsX (PDB ID: 6A1K) [36], *Aquifex aeolicus* PlsY (PDB ID: 5XJ9) [34], and *Thermotoga maritima* PlsC (PDB ID: 5KYM) [35]. Figure created using BioRender.com.

Phosphatidic acid is the central intermediate of phospholipid synthesis and the most widely distributed pathway for phospholipid synthesis in bacteria is the PlsXYC pathway [17,29,30,31,32]. The first step is catalyzed by acyl-ACP:phosphate transacylase (PlsX), which catalyzes the reversible conversion of acyl-ACP from FASII to acyl phosphate. The second step is catalyzed by glycerol-phosphate acyltransferase (PlsY), which utilizes acyl phosphate to convert glycerol-3-phosphate to lysophosphatidic acid. The third step is catalyzed by 1-acyl-sn-glycerol-3-phosphate acyltransferase (PlsC), which utilizes acyl-ACP to convert lysophosphatidic acid to phosphatidic acid. Fatty acid kinase is a two-component system consisting of a fatty acid binding protein (FakB) that obtains eFA from the bilayer and presents the eFA to the kinase (FakA) for phosphorylation to acyl phosphate, and then FakB exchanges the acyl phosphate with eFA in the bilayer and the cycle repeats [33]. The acyl phosphates made by FakAB can be used by PlsY or converted to acyl-ACP by PlsX for FASII modification and/or PlsC utilization (Figure 1). Thus, through PlsX, FakAB products can be used for both steps of phospholipid synthesis. PlsY is an integral membrane protein with a seven-transmembrane helix fold [34], and PlsC is a monotopic integral membrane protein anchored by a hydrophobic/aromatic amino-terminal two-helix motif [35] (Figure 2). PlsX and FakB are soluble proteins that must solve the topological problem of exchanging their insoluble substrates with the membrane, and recent studies have shed light onto these processes. The FakAB system is a Gram-positive strategy to activate eFA for cell metabolism, whereas Gram-negative bacteria generally use acyl-CoA and acyl-ACP synthetases to activate eFA.

### 2.2. Acyl-ACP:Phosphate Transacylase (PlsX)

Immunofluorescent imaging of cell fractionation experiments using a PlsX antibody found PlsX associated with the membrane in intact *Bacillus subtilis* cells, but when *B. subtilis* cells were disrupted then PlsX was found in the soluble fraction [31]. Fluorescence microscopy of *B. subtilis* cells expressing green fluorescent protein (GFP) fused to PlsX showed PlsX accumulation at membrane foci during early log phase growth, followed by uniform distribution on the membrane during later stage growth [37]. These dynamic subcellular localization experiments implicated PlsX as a peripheral membrane protein that can reversibly associate with the membrane.

**Figure 3:**
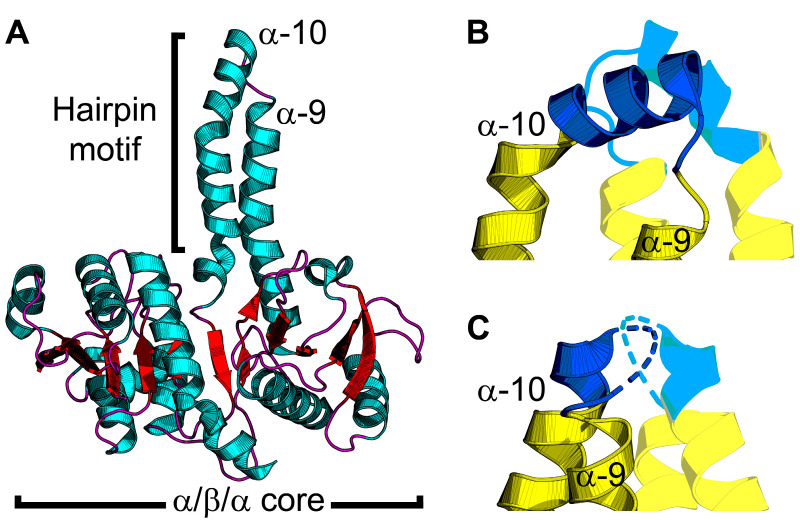
PlsX structure and conformational changes. (**A**) The PlsX protomer contains an α/β/α sandwich core domain and protruding hairpin motif made by α-helices α-9 and α-10. PlsX is colored by secondary structure elements with teal α-helices and red β-strands and loops. (**B**,**C**) Zoomed-in views of the tip of the hairpin motif. PlsX is dimeric and the hairpin motif of the opposite protomer is shown with transparency. The membrane-binding segment is shown in blue and the soluble segment is yellow. (**B**) The helix conformation of the hairpin motif is proposed to be the membrane-bound conformation. (**C**) The loop conformation of the hairpin motif is proposed to be the cytosolic conformation. The structures in (**A**,**B**) are from *Bacillus subtilis* PlsX with the hairpin tip in the helix conformation (PDB ID: 6A1K) [36] and the structure in (**C**) is from *Enterococcus faecalis* PlsX with the hairpin tip in the loop conformation (PDB ID: 1U7N) [39].

PlsX forms a soluble dimer, and the crystal structure shows that each protomer contains a core α/β/α sandwich resembling a Rossmann fold, and an α-helical hairpin motif that extends away from the core domain (Figure 3A) [38]. The hairpins from each protomer, made up of helices α-9 and α-10 connected by a loop, combine to make an amphipathic helical bundle (Figure 3B) [39]. The loop at the tip of the hairpin contains hydrophobic residues needed for lipid binding. PlsX co-sediments with liposomes made from *B. subtilis* membranes, but loses the lipid binding property when residues in the hydrophobic loop are mutated to glutamates [40]. Fluorescence microscopy of *B. subtilis* cells expressing GFP fused to PlsX containing glutamate in the hairpin loop showed cytosolic accumulation of PlsX, confirming this region is essential for membrane association in vivo [40]. *B. subtilis* cells expressing PlsX containing glutamate in the hairpin have a growth defect, indicating that membrane association is necessary for function in vivo [40]. 

Direct SPR measurements of PlsX–lipid binding confirmed that PlsX binds anionic phosphatidylglycerol with nanomolar affinity but does not bind zwitterionic phosphatidylcholine [41]. EPR analysis of PlsX interacting with spin-labeled lipids showed spectral perturbation from the lipid headgroup to the center of the bilayer [41]. SPR and EPR analysis of PlsX containing glutamate in the hairpin loop showed that the mutant PlsX still bound phosphatidylglycerol but only elicited minimal spectral perturbation at the lipid head group region, suggesting only superficial membrane association [41]. These data indicate that PlsX inserts into a membrane leaflet. PlsX was also crystallized in the presence of a product analog (palmitoyl phosphoramide) and although the analog was not resolved in the final structure, a new conformation of the hairpin loop was observed (Figure 3C) [36]. In this structure, the hairpin loop adopts an amphipathic α-helix conformation, and introduction of polar interfacial residues to disrupt the amphipathicity of this segment caused cytosolic accumulation of GFP-fused PlsX [36]. Mutations that compromised membrane association from this segment also correlated with growth defects [36], which agrees with a similar report [40]. These data suggest a model for PlsX membrane binding where the hairpin loop (Figure 3C) undergoes a conformational change at the membrane surface to become an amphipathic α-helix (Figure 3B) that inserts into the bilayer to transfer acyl phosphate to PlsY or extract acyl phosphate from the membrane. Additionally, the catalytic site is inferred to be located at the interface between the PlsX protomers, but the only structures available are ligand-free. A PlsX-acyl phosphate complex structure would validate this site, help clarify the catalytic mechanism, and determine the effect acyl phosphate binding has on the hairpin and membrane binding. It is still unclear if PlsX ever comes off the membrane in vivo and what steps in catalysis, if any, require membrane disengagement. Where is the acyl-ACP binding site and how does binding this substrate affect membrane binding? Docking calculations predict an acyl-ACP binding site on a positively charged surface patch on the opposite side of the hairpin tip [41]. This binding mode would enable PlsX to interact with acyl-ACP without disengaging from the membrane bilayer; however, direct binding experiments are needed to validate this prediction. How does acyl phosphate binding affect membrane binding? PlsX–membrane association is sensitive to lipid unsaturation [41] and may also be sensitive to changes in the membrane curvature elastic stress imposed by acyl phosphate in the bilayer. A combination of SPR and EPR could be helpful in clarifying the mechanism with this granularity.

### 2.3. Fatty Acid Kinase (FakAB)

The FakAB system in bacteria typically consists of one FakA and multiple FakBs to expand the spectrum of eFAs that can be utilized and establish FakB as the eFA selectivity filter. FakA forms a soluble dimer with a unique topology of a zinc finger-containing domain flanked by an amino terminal DhaL domain and carboxy terminal DegV domain [42,43]. The FakA domains can be independently expressed and purified, but there is no evidence that FakA is an interfacial enzyme or any of the domains contact the membrane bilayer. *S. aureus* encodes two FakB genes and biochemical assays show that FakB1 selectively binds the saturated fatty acids myristate and palmitate, and FakB2 selectivity binds the monounsaturated fatty acid oleate [33]. The Gram-positive pathogen *Streptococcus pneumoniae* encodes three FakBs, and FakB3 can bind polyunsaturated fatty acid linoleate, linoleneate, and arachidonate in a biochemical assay [44].

The available FakB crystal structures all contain bound fatty acid since ligand-free FakB is insoluble [45]. It is unclear why ligand-free FakB is insoluble, and it could require lipid or detergent for stability. Cell compartment localization experiments have not been carried out with FakAB. The fatty acid length that FakB can accommodate is determined by an amino acid ruler at the end of the hydrophobic binding pocket, whereas fatty acid saturation selectivity arises from differences in the binding pocket shape. These differences do not impact the protein surface, so all FakBs can still interact with FakA. 

**Figure 4:**
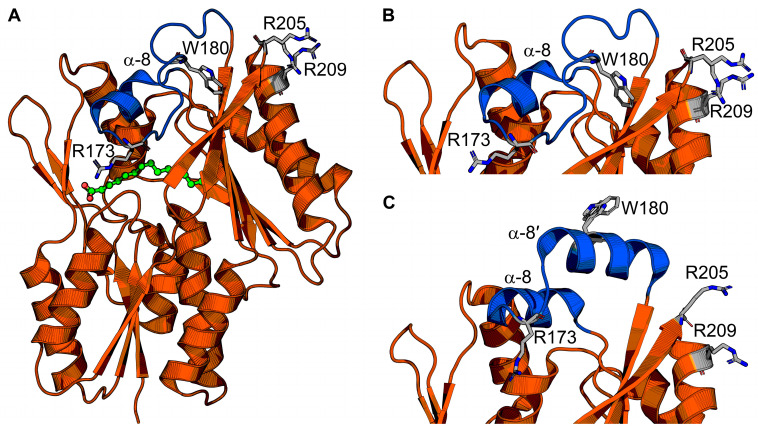
FakB structure and conformational changes. (**A**) The FakB structure envelopes the fatty acid (green). A cap (blue) closes the fatty acid binding site and is the membrane-binding segment. The soluble segment is orange. Cap amino acid R173 engages the fatty acid and seals the fatty acid binding site from bulk solvent. Amino acids W180, R205, and R209 are residues that bind or penetrate the membrane. (**B,C**) Zoomed-in views of the helix–loop “closed” cap conformation that is proposed to be the cytosolic conformation (**B**), and the helix–loop–helix “open” cap conformation that is proposed to be the membrane-bound conformation (**C**). The structures in (**A**,**B**) are from *Staphylococcus aureus* FakB1 with a “closed” cap (PDB ID: 6ALW) [45], and the structure in (**C**) is from *Staphylococcus aureus* FakB1 with an “open” cap (PDB ID: 6MH9) [16].

The FakB structure is monomeric and consists of an amino terminal EDD fold domain and carboxy terminal six-stranded β-sheet flanked by α-helices that binds fatty acid at the interface between the domains (Figure 4A) [43,45]. The crystal structure of FakB1 shows that the fatty acid binding site is a slightly curved tunnel with an isoleucine ruler that cannot accommodate the kink of a fatty acid with a cis-double bond [45]. The crystal structure of FakB2 shows that phenylalanine and isoleucine side chains in the fatty acid binding site create a sharp turn in the tunnel at the cis-9 double bond position to accommodate the kink of monounsaturated fatty acids [46]. The crystal structure of FakB3 shows an expanded fatty acid binding site that can accommodate multiple kinks from the multiple cis-double bonds in polyunsaturated fatty acids [44]. In all cases, the fatty acid binding site is covered by a helix–loop “closed” cap made from helix α-8 surrounded by an electropositive surface (Figure 4B).

**Figure 5:**
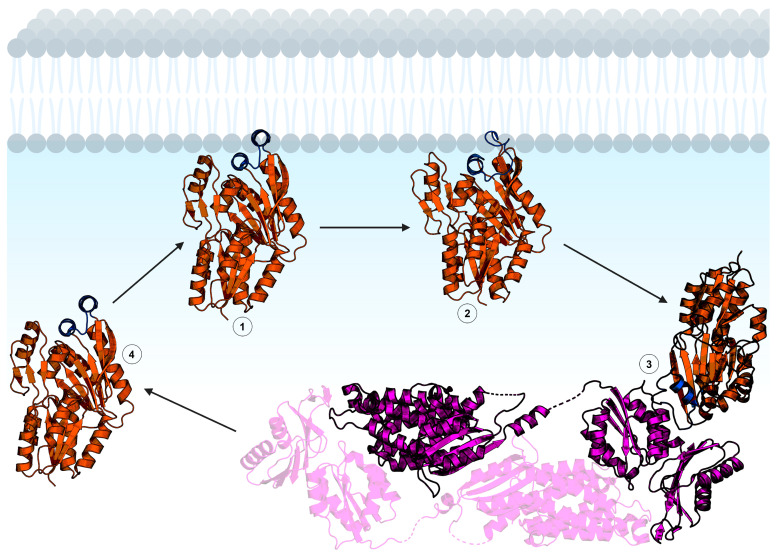
Hypothetical mechanism of FakB membrane binding. The FakB helix–loop–helix cap motif membrane binding domain is shown in blue and the soluble domain is orange. 1: FakB with an “open” cap scans the membrane for a fatty acid. 2: Upon binding fatty acid, the FakB cap transitions to the “closed” conformation. 3: FakB disengages from the membrane and binds FakA while in the “closed” cap conformation. FakA (magenta) is dimeric and the opposite protomer is shown with transparency. 4: After catalysis, FakB disengages from FakA in the “open” cap conformation to return to the membrane, deposit the acyl phosphate in the bilayer, and search for another fatty acid. The structures shown are *Staphylococcus aureus* FakB1 with an “open” cap (PDB ID: 6MH9) [16], *Staphylococcus aureus* FakB1 with a “closed” cap (PDB ID: 6ALW) [45], and *Streptococcus suis* FakAB2 complex (PDB ID: 7W7H) [43]. Figure created using BioRender.com.

Liposome sedimentation experiments show FakB1 sediments with anionic phosphatidylglycerol but not zwitterionic phosphatidylcholine, and SPR direct measurements of FakB1–lipid binding measured micromolar affinity for phosphatidylglycerol and no binding to phosphatidylcholine [16]. FakB also has micromolar affinity for FakA [46]. The crystal structure of an FakB1 mutant (A121I) captured an “open” conformation of the cap where a portion of the loop forms a new amphipathic α-helix (helix α-8′) that rotates away from the protein and makes a helix–loop–helix cap that exposes the fatty acid binding site (Figure 4C) [16]. NMR analysis of FakB1 determined that the cap is dynamic in solution and MD simulations of FakB1 with an accelerated membrane model and full membrane/full lipid model of phosphatidylglycerol predicted that the new amphipathic α-helix of the “open” cap inserts below the phosphate plane of the bilayer [16]. These structural transitions are thought to create a diffusion channel for the hydrophobic fatty acid tail to access the hydrocarbon core and place the carboxyl group at the phosphate layer [16]. Mutagenesis of key amino acid side chains R205, R209, and W180 in the cap region that are predicted to insert into the membrane yield enzymes that do not bind phospholipid liposomes or catalyze the FakB1 reaction when substrate is presented in phosphatidylglycerol liposomes [16]. Cell localization experiments could be an approach to determine if FakB recruits FakA to the membrane for protein–protein interactions and catalysis at the cell membrane, or if FakB disengages from the membrane after fatty acid binding. The FakB–membrane affinity was measured with fatty acid-bound FakB and is log-fold weaker than PlsX. FakB disengagement from the membrane is likely needed to interact with FakA because site-directed mutagenesis experiments on the FakB cap implicate the cap in binding both the cell membrane and FakA. Coupled biochemical assays using FakAB, PlsX, and ACP suggest FakB can deliver fatty acid to PlsX, thus bypassing the need for a membrane [43], but the molecular mechanism of how that would work has not been determined. If FakB does disengage from the membrane after binding fatty acid in the “closed” cap conformation, then the acyl phosphate made by FakA could trigger the “open” cap conformation and enhance membrane affinity (Figure 5). NMR could be useful to compare the cap dynamics of fatty acid-bound FakB with acyl phosphate-bound FakB to better understand how the fatty acid binding site communicates with the cap. SPR and EPR could be used to study the impact of acyl phosphate on FakB–membrane association. 

## 3. Conclusions

Interfacial enzymes play a significant role in the bacterial strategy to eat environmental fatty acids, from their activation to making them compatible with the cell physiology program. Converting eFA nutrients to acyl-ACP enables their theoretical utilization in other biological processes, in addition to phospholipid synthesis such as polyketide synthesis and lipoprotein synthesis. The work discussed in this review presents current advances in understanding how key interfacial enzymes involved in eFA metabolism interact with the membrane. Studying PlsX and FakB adds to the molecular mechanistic understanding of how peripheral membrane proteins use conformational changes to precisely regulate their activation, localization, and integration into the membrane. More studies must be carried out to understand how these enzymes operate in their functional environment—the membrane. A major advance would be the structure of an interfacial enzyme embedded in the membrane. Complex visualization of an enzyme integrating with the membrane bilayer to extract or deposit its cargo would both enhance our biological understanding of this process and refine our computational capabilities to simulate these phenomena more accurately.

## 4. Discussion

Peripheral membrane proteins are readily purified from the cytosolic fraction of cells, which makes them suitable for detailed mechanistic studies; however, there is limited protein–membrane structural information because the amino acid sequences of their membrane binding segments are non-obvious and membrane binding studies are often omitted from their characterizations. Bioinformatic tools and workflows have been developed to predict the membrane-binding domains of modular proteins. These tools can be deployed to generate testable hypotheses about the structural elements that drive interfacial enzyme–membrane binding and add to this knowledgebase. There are also thousands of protein structures in the Protein Data Bank with the annotation “unknown function” and these tools could potentially identify peripheral membrane proteins among them. The protocol developed by Bhardwaj et al. [47] uses a support vector machine to classify proteins by net charge, distribution of cationic patches, and amino acid composition. This protocol predicts membrane binders based on the expectation that electrostatic complementarity between cationic proteins and anionic membranes is a major driver of binding. In a test set of 40 known membrane proteins and 230 non-binding proteins, the protocol was ~90% accurate in predicting membrane binding properties, and in a small sample of four structurally related C2 domains with unknown membrane binding properties, the protocol correctly predicted the single membrane binder that was confirmed by SPR. Another useful tool is the Drugging pRotein mEmbrAne Machine learning Method (DREAMM; https://dreamm.ni4os.eu/ (accessed on 1 April 2023)), which extracts physicochemical and biochemical information from a three-dimensional protein structure and predicts membrane-penetrating amino acids [48,49]. In a dataset of 54 known peripheral membrane proteins with known three-dimensional structures and experimentally known membrane-penetrating amino acids, DREAMM correctly predicted membrane-penetrating amino acids with a macro-averaged *F*_1_ score = 0.92 and MCC = 0.84. In an independent test set with experimentally known protein–membrane regions, DREAMM demonstrated 91% precision in identifying membrane-penetrating amino acids. Bioinformatics tools like these will help advance understanding of the complexity of lipid–protein interactions at cell membranes by enabling the major step of identification of protein–membrane interaction sites of peripheral membrane proteins.

## Data Availability

Coordinates and structure factors for the structures discussed in this review are freely accessibility from the Protein Data Bank (www.rcsb.org (accessed on 1 March 2023)) using the PDB ID accession codes.

## References

[1] Strahl H., Errington J. (2017). Bacterial membranes: Structure, domains, and function. Annu. Rev. Microbiol..

[2] Lu G., Xu Y., Zhang K., Xiong Y., Li H., Cui L., Wang X., Lou J., Zhai Y., Sun F. (2017). Crystal structure of *E. coli* apolipoprotein N-acyl transferase. Nat. Commun..

[3] Gardiner J.H.t., Komazin G., Matsuo M., Cole K., Gotz F., Meredith T.C. (2020). Lipoprotein N-Acylation in *Staphylococcus aureus* Is Catalyzed by a Two-Component Acyl Transferase System. mBio.

[4] Chen X., Alonzo F. (2019). Bacterial lipolysis of immune-activating ligands promotes evasion of innate defenses. Proc. Natl. Acad. Sci. USA.

[5] Seelig J. (2004). Thermodynamics of lipid-peptide interactions. Biochim. Biophys. Acta.

[6] Whited A.M., Johs A. (2015). The interactions of peripheral membrane proteins with biological membranes. Chem. Phys. Lipids.

[7] Malmberg N.J., Van Buskirk D.R., Falke J.J. (2003). Membrane-docking loops of the cPLA2 C2 domain: Detailed structural analysis of the protein-membrane interface via site-directed spin-labeling. Biochemistry.

[8] Gamsjaeger R., Johs A., Gries A., Gruber H.J., Romanin C., Prassl R., Hinterdorfer P. (2005). Membrane binding of β2-glycoprotein I can be described by a two-state reaction model: An atomic force microscopy and surface plasmon resonance study. Biochem. J..

[9] Verma M.L., Azmi W., Kanwar S.S. (2008). Microbial lipases: At the interface of aqueous and non-aqueous media. A review. Acta Microbiol. Immunol. Hung..

[10] Del Vecchio K., Stahelin R.V. (2016). Using Surface Plasmon Resonance to Quantitatively Assess Lipid-Protein Interactions. Methods Mol. Biol..

[11] Sahu I.D., Lorigan G.A. (2020). Electron paramagnetic resonance as a tool for studying membrane proteins. Biomolecules.

[12] Smirnova T.I., Smirnov A.I. (2015). Peptide-membrane interactions by spin-labeling EPR. Methods Enzymol..

[13] Opella S.J., Marassi F.M. (2017). Applications of NMR to membrane proteins. Arch. Biochem. Biophys..

[14] Oxenoid K., Chou J.J. (2016). A functional NMR for membrane proteins: Dynamics, ligand binding, and allosteric modulation. Protein Sci..

[15] Pant S., Tajkhorshid E. (2020). Microscopic characterization of GRP1 PH domain interaction with anionic membranes. J. Comput. Chem..

[16] Gullett J.M., Cuypers M.G., Grace C.R., Pant S., Subramanian C., Tajkhorshid E., Rock C.O., White S.W. (2022). Identification of structural transitions in bacterial fatty acid binding proteins that permit ligand entry and exit at membranes. J. Biol. Chem..

[17] Parsons J.B., Rock C.O. (2013). Bacterial lipids: Metabolism and membrane homeostasis. Prog. Lipid Res..

[18] White S.W., Zheng J., Zhang Y.-M., Rock C.O. (2005). The structural biology of type II fatty acid biosynthesis. Annu. Rev. Biochem..

[19] Radka C.D., Rock C.O. (2022). Mining fatty acid biosynthesis for new antimicrobials. Annu. Rev. Microbiol..

[20] Wittke F., Vincent C., Chen J., Heller B., Kabler H., Overcash J.S., Leylavergne F., Dieppois G. (2020). Afabicin, a first-in-class antistaphylococcal antibiotic, in the treatment of acute bacterial skin and skin structure infections: Clinical noninferiority to vancomycin/linezolid. Antimicrob. Agents Chemother..

[21] Vuong C., Yeh A.J., Cheung G.Y., Otto M. (2016). Investigational drugs to treat methicillin-resistant *Staphylococcus aureus*. Expert Opin. Investig. Drugs.

[22] Quehenberger O., Dennis E.A. (2011). The human plasma lipidome. N. Engl. J. Med..

[23] Radka C.D., Batte J.L., Frank M.W., Rosch J.W., Rock C.O. (2021). Oleate hydratase (OhyA) is a virulence determinant in *Staphylococcus aureus*. Microbiol. Spectr..

[24] Brinster S., Lamberet G., Staels B., Trieu-Cuot P., Gruss A., Poyart C. (2009). Type II fatty acid synthesis is not a suitable antibiotic target for Gram-positive pathogens. Nature.

[25] Morvan C., Halpern D., Kenanian G., Pathania A., Anba-Mondoloni J., Lamberet G., Gruss A., Gloux K. (2017). The *Staphylococcus aureus* FASII bypass escape route from FASII inhibitors. Biochimie.

[26] Kenanian G., Morvan C., Weckel A., Pathania A., Anba-Mondoloni J., Halpern D., Gaillard M., Solgadi A., Dupont L., Henry C. (2019). Permissive fatty acid incorporation promotes staphylococcal adaptation to FASII antibiotics in host environments. Cell Rep..

[27] Balemans W., Lounis N., Gilissen R., Guillemont J., Simmen K., Andries K., Koul A. (2010). Essentiality of FASII pathway for *Staphylococcus aureus*. Nature.

[28] Antimicrobial Resistance C. (2022). Global burden of bacterial antimicrobial resistance in 2019: A systematic analysis. Lancet.

[29] Lu Y.-J., Zhang Y.-M., Grimes K.D., Qi J., Lee R.E., Rock C.O. (2006). Acyl-phosphates initiate membrane phospholipid synthesis in Gram-positive pathogens. Mol. Cell.

[30] Yao J., Rock C.O. (2013). Phosphatidic acid synthesis in bacteria. Biochim. Biophys. Acta.

[31] Paoletti L., Lu Y.-J., Schujman G.E., de Mendoza D., Rock C.O. (2007). Coupling of fatty acid and phospholipid synthesis in *Bacillus subtilis*. J. Bacteriol..

[32] Zhang Y.-M., Rock C.O. (2008). Acyltransferases in bacterial glycerophospholipid synthesis. J. Lipid Res.

[33] Parsons J.B., Broussard T.C., Bose J.L., Rosch J.W., Jackson P., Subramanian C., Rock C.O. (2014). Identification of a two-component fatty acid kinase responsible for host fatty acid incorporation by *Staphylococcus aureus*. Proc. Natl. Acad. Sci. USA.

[34] Li Z., Tang Y., Wu Y., Zhao S., Bao J., Luo Y., Li D. (2017). Structural insights into the committed step of bacterial phospholipid biosynthesis. Nat. Commun..

[35] Robertson R.M., Yao J., Gajewski S., Kumar G., Martin E.W., Rock C.O., White S.W. (2017). A two-helix motif positions the lysophosphatidic acid acyltransferase active site for catalysis within the membrane bilayer. Nat. Struct. Mol. Biol..

[36] Jiang Y., Dai X., Qin M., Guo Z. (2019). Identification of an amphipathic peptide sensor of the *Bacillus subtilis* fluid membrane microdomains. Commun. Biol..

[37] Sastre D.E., Bisson-Filho A., de Mendoza D., Gueiros-Filho F.J. (2016). Revisiting the cell biology of the acyl-ACP: Phosphate transacylase PlsX suggests that the phospholipid synthesis and cell division machineries are not coupled in *Bacillus subtilis*. Mol. Microbiol..

[38] Badger J., Sauder J.M., Adams J.M., Antonysamy S., Bain K., Bergseid M.G., Buchanan S.G., Buchanan M.D., Batiyenko Y., Christopher J.A. (2005). Structural analysis of a set of proteins resulting from a bacterial genomics project. Proteins.

[39] Kim Y., Li H., Binkowski T.A., Holzle D., Joachimiak A. (2009). Crystal structure of fatty acid/phospholipid synthesis protein PlsX from *Enterococcus faecalis*. J. Struct. Funct. Genomics.

[40] Sastre D.E., Pulschen A.A., Basso L.G.M., Benites Pariente J.S., Marques Netto C.G.C., Machinandiarena F., Albanesi D., Navarro M., de Mendoza D., Gueiros-Filho F.J. (2020). The phosphatidic acid pathway enzyme PlsX plays both catalytic and channeling roles in bacterial phospholipid synthesis. J. Biol. Chem..

[41] Sastre D.E., Basso L.G.M., Trastoy B., Cifuente J.O., Contreras X., Gueiros-Filho F., de Mendoza D., Navarro M., Guerin M.E. (2020). Membrane fluidity adjusts the insertion of the transacylase PlsX to regulate phospholipid biosynthesis in Gram-positive bacteria. J. Biol. Chem..

[42] Subramanian C., Cuypers M.G., Radka C.D., White S.W., Rock C.O. (2022). Domain architecture and catalysis of the *Staphylococcus aureus* fatty acid kinase. J. Biol. Chem..

[43] Shi Y., Zang N., Lou N., Xu Y., Sun J., Huang M., Zhang H., Lu H., Zhou C., Feng Y. (2022). Structure and mechanism for streptococcal fatty acid kinase (Fak) system dedicated to host fatty acid scavenging. Sci. Adv..

[44] Gullett J.M., Cuypers M.G., Frank M.W., White S.W., Rock C.O. (2019). A fatty acid-binding protein of *Streptococcus pneumoniae* facilitates the acquisition of host polyunsaturated fatty acids. J. Biol. Chem..

[45] Cuypers M.G., Subramanian C., Gullett J.M., Frank M.W., White S.W., Rock C.O. (2019). Acyl chain selectivity and physiological roles of *Staphylococcus aureus* fatty acid binding proteins. J. Biol. Chem..

[46] Broussard T.C., Miller D.J., Jackson P., Nourse A., White S.W., Rock C.O. (2016). Biochemical roles for conserved residues in the bacterial fatty acid binding protein family. J. Biol. Chem..

[47] Bhardwaj N., Stahelin R.V., Langlois R.E., Cho W., Lu H. (2006). Structural bioinformatics prediction of membrane-binding proteins. J. Mol. Biol..

[48] Chatzigoulas A., Cournia Z. (2022). Predicting protein-membrane interfaces of peripheral membrane proteins using ensemble machine learning. Brief. Bioinform..

[49] Chatzigoulas A., Cournia Z. (2022). DREAMM: A web-based server for drugging protein-membrane interfaces as a novel workflow for targeted drug design. Bioinformatics.

